# Inflammatory biomarkers and therapeutic potential of milk exosome-mediated CCL7 siRNA in murine intestinal ischemia-reperfusion injury

**DOI:** 10.3389/fimmu.2024.1513196

**Published:** 2025-01-20

**Authors:** WenDong Chen, WenPing Xu, Li Ma, Chun Bi, MeiXu Yang, Wei Yang

**Affiliations:** Department of Anesthesiology, First Affiliated Hospital of Kunming Medical University, Kunming, Yunnan, China

**Keywords:** intestinal ischemia-reperfusion injury, biomarkers, inflammation, CCL7, milk-derived exosomes, siRNA delivery

## Abstract

**Background:**

Intestinal ischemia-reperfusion injury (IIRI) is a severe clinical condition associated with high morbidity and mortality. Despite advances in understanding the pathophysiology of IIRI, effective diagnostic and therapeutic strategies remain limited.

**Methods:**

Using transcriptome sequencing in a mouse model of IIRI, we identified potential biomarkers that were significantly upregulated in the IIRI group compared to the sham group. Based on these findings, we developed and evaluated a therapeutic strategy using milk-derived exosomes loaded with siRNA targeting CCL7 (M-Exo/siCCL7).

**Results:**

Focusing on Ccl7 as a hub gene, we explored the therapeutic efficacy of milk-derived exosomes loaded with siRNA targeting Ccl7 (M-Exo/siCCL7) in the IIRI model. M-Exo/siCCL7 treatment effectively attenuated intestinal inflammation and injury, as evidenced by reduced histological damage, decreased serum markers of intestinal barrier dysfunction, and attenuated systemic inflammation.

**Conclusion:**

Our findings provide new insights into the molecular mechanisms underlying IIRI, identify potential diagnostic biomarkers, and highlight the promise of exosome-based siRNA delivery as a novel therapeutic approach for IIRI.

## 1 Introduction

Intestinal ischemia-reperfusion injury (IIRI) is a severe clinical complication associated with significant morbidity and mortality (1). It can occur in various conditions such as shock, abdominal trauma, intestinal obstruction, organ transplantation, and extracorporeal circulation (2, 3). The intestine is particularly vulnerable to ischemia-reperfusion injury compared to other organs (4). During IIRI, the restoration of blood flow after a period of ischemia paradoxically exacerbates the tissue damage initiated during the ischemic period (5, 6). The pathophysiology of IIRI involves a complex interplay of multiple factors, including oxidative stress, inflammatory response, and cell apoptosis (7, 8). Currently, there is a lack of effective diagnostic methods and therapeutic approaches for IIRI. Therefore, it is crucial to identify key biomarkers and explore novel treatment strategies for IIRI.

With the advancement of high-throughput sequencing technologies, transcriptome analysis has become a powerful tool to investigate the molecular mechanisms underlying various diseases (9). Several studies have utilized RNA sequencing to identify differentially expressed genes (DEGs) and key pathways involved in IIRI (10, 11). These findings provide valuable insights into the pathogenesis of IIRI and potential therapeutic targets. However, further validation and functional studies are needed to translate these findings into clinical applications.

Animal models play a vital role in studying the mechanisms of IIRI and evaluating the efficacy of therapeutic interventions (3, 10). Among various animal models, the mouse IIRI model has been widely used due to its similarities to human intestinal physiology and the availability of genetic tools (11). By inducing ischemia-reperfusion injury in the mouse intestine, researchers can investigate the molecular events and cellular responses during IIRI (12). Moreover, the mouse IIRI model allows for the screening and validation of potential biomarkers and therapeutic targets (11).

RNA interference (RNAi) has emerged as a promising approach for gene silencing and targeted therapy (13, 14). Small interfering RNAs (siRNAs) are short double-stranded RNA molecules that can specifically degrade complementary mRNA sequences, leading to gene silencing (12). However, the delivery of siRNAs to target tissues remains a major challenge due to their instability and poor cellular uptake. Exosomes, which are nanoscale extracellular vesicles, have been explored as natural carriers for siRNA delivery (15). Milk-derived exosomes (M-Exos) have attracted particular attention due to their biocompatibility, stability, and ability to cross biological barriers (16–18). Recent studies have demonstrated the potential of M-Exos as a delivery vehicle for siRNAs in the treatment of inflammatory bowel disease (19). This suggests that M-Exo-mediated siRNA delivery could also be a promising strategy for treating IIRI.

**Figure 1:**
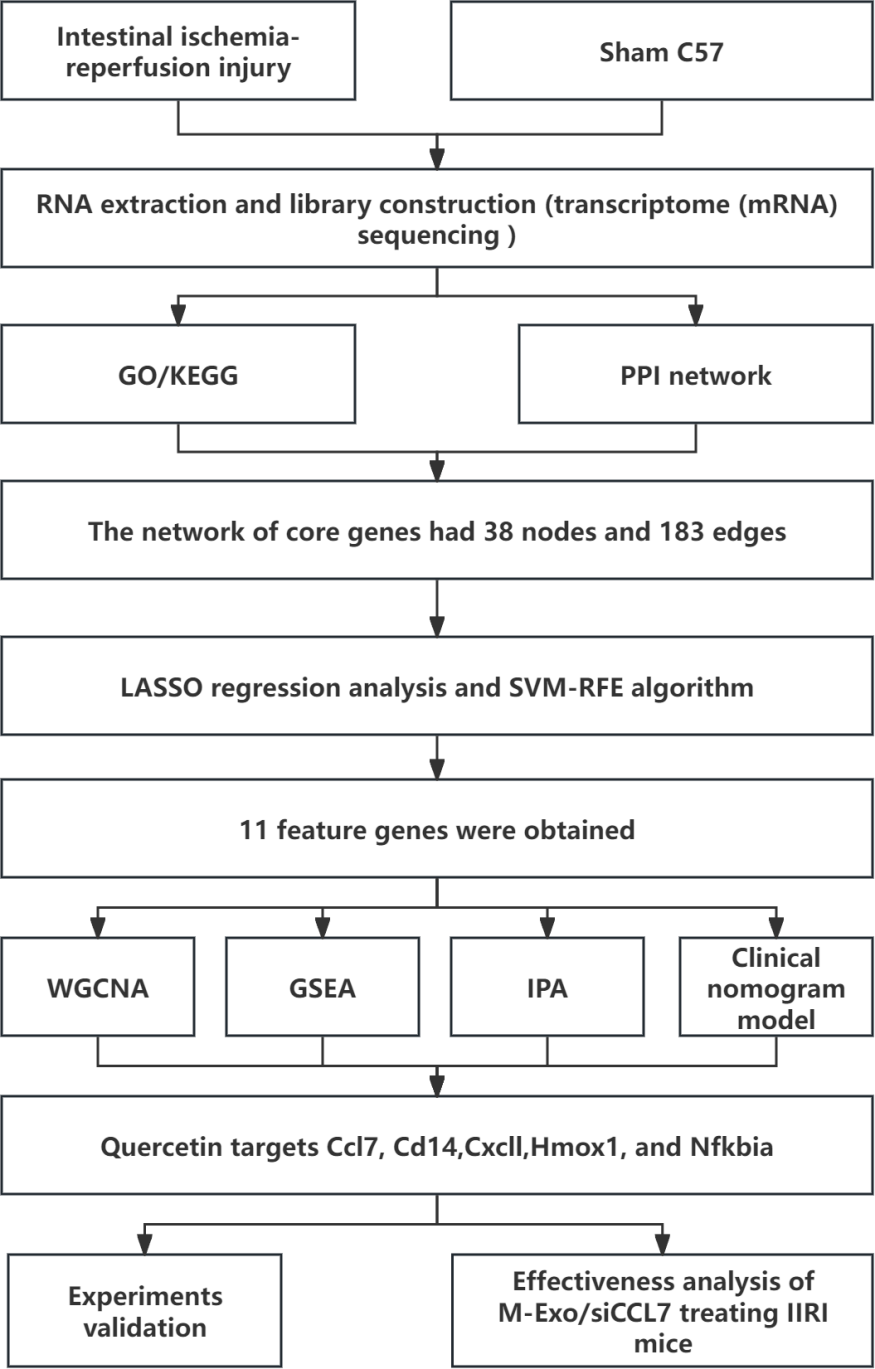
Schematic overview of the study design. The workflow includes transcriptome sequencing of intestinal tissues from sham and IIRI mice, bioinformatics analyses (GO, KEGG, PPI, WGCNA, LASSO, SVM-RFE, GSEA, and IPA), validation of identified biomarkers (Ccl7, Cd14, Cxcl1, Hmox1, and Nfkbia) by qPCR and IHC, and evaluation of the therapeutic efficacy of milk exosome-mediated siCCL7 delivery in the IIRI model.

In this study, we aimed to identify key biomarkers and explore the therapeutic potential of M-Exo-mediated siRNA delivery in IIRI using a mouse model (**Figure 1**). We performed RNA sequencing on intestinal tissues from sham and IIRI mice to identify DEGs and key pathways involved in IIRI. Bioinformatics analyses, including weighted gene co-expression network analysis (WGCNA), protein-protein interaction (PPI) network analysis, and machine learning algorithms, were employed to screen for potential biomarkers. The expression levels of the identified biomarkers were validated using quantitative PCR and immunohistochemistry. Furthermore, we investigated the therapeutic efficacy of M-Exo-mediated delivery of siRNA targeting a selected biomarker in the mouse IIRI model. Our findings provide new insights into the molecular mechanisms of IIRI and highlight the potential of M-Exo-mediated siRNA delivery as a novel therapeutic approach for IIRI.

## 2 Materials and methods

### 2.1 RNA extraction and library construction

#### 2.1.1 Sample preparation

The Experimental Animal Center at Kunming Medical University provided 70 healthy male mice of the C57BL/6J strain (6-8 weeks old, weighing 18-22 g) (Certificate of Conformity: SCXK2019-0008). The Ethics Committee of Kunming Medical University granted consent for the use of animals in research (approval number: KMMU20220927), and all methods followed the guidelines set by the National Institutes of Health for the use of laboratory animals. The research has been conducted according to the principles stated in the ARRIVE guidelines. Animals were kept in captivity for two weeks prior to the start of the experiment. They were maintained at a temperature of 25°C and 50 mL/L humidity, ensuring an environment with a 12 h cycle of light and darkness. Additionally, they were provided with an adequate supply of food and water. The mice were fasted for 12 h before the experiments. The NIH Guide for the Care and Use of Laboratory Animals was followed for all animal research, and all experiments were designed to minimize pain and distress for the animals. We adhere to all applicable federal regulations regarding the use and treatment of animals.

### 2.2 Animal grouping

The 60 mice were divided into two cohorts: a target screening cohort (n = 40) for identifying key targets through sequencing and bioinformatics analysis, and a treatment validation cohort (n = 30) for validating the therapeutic effects of the identified targets.

In the target screening cohort, mice were randomly assigned to the IIRI and sham-operated groups (Sham). In the treatment validation cohort, mice were randomly divided into three groups (n = 10 per group): (1) Sham group: Mice received intraperitoneal injections of saline for 7 days before undergoing sham surgery. (2) Model group: Mice received intraperitoneal injections of saline for 2 consecutive days, followed by the induction of the II/R model. (3) Treatment group: Before II/R surgery, mice were pretreated with M-Exo/siCCL7 (milk-derived exosomes loaded with siCCL7) via gavage, once daily for 2 consecutive days.

### 2.3 IIRI model construction

Mice were anesthetized with sodium pentobarbital (50 mg/kg, intraperitoneal injection). After confirming adequate anesthesia, a midline laparotomy was performed to expose the superior mesenteric artery (SMA). The SMA was occluded using a microvascular clip for 30 minutes. During this period, the abdomen was temporarily closed with surgical clips to prevent fluid loss. After 30 minutes of ischemia, the abdomen was reopened, the vascular clip removed to allow reperfusion, and the incision was permanently closed. The tissue was then allowed to reperfuse for 2 hours before euthanasia. The same operation was performed on mice in the sham-operated group, with the exception of superior jejunal segment mesenteric artery blockage (11). For all analyses, the jejunal segment (10 cm distal to the ligament of Treitz) was used, as this region shows consistent ischemic changes and minimal collateral circulation. This standardization was maintained across all experimental procedures to ensure consistency. After 2 hours of reperfusion and prior to tissue collection, we documented the gross anatomical changes in the intestine.

### 2.4 RNA sequencing of murine small intestine tissues in ischemia-reperfusion injury

Mice were euthanized by sodium pentobarbital overdose injection after 2 hours of reperfusion, and the small intestine tissue was extracted by opening the abdomen. After being thoroughly cleaned, a small portion of the small intestine was quickly frozen in liquid nitrogen and transferred to a -80°C refrigerator for gene sequencing.

The organization obtained 40 samples of C57BL/6J mouse small intestine tissue, including 20 normal samples and 20 samples with IIRI. Following the manufacturer’s instructions, total RNA was extracted using the TRIzol reagent (Invitrogen, Carlsbad, CA, USA). We quantified the amount and purity of RNA in all samples using the NanoDrop ND-1000 (NanoDrop, Wilmington, DE, USA). The RNA integrity was assessed using the Bioanalyzer 2100 (Agilent, CA, USA) and validated using denaturing agarose gel electrophoresis. RNA integrity was assessed using the Agilent 2100 Bioanalyzer (Agilent Technologies, Santa Clara, CA, USA). Samples with an RNA Integrity Number (RIN) ≥ 7.0 were considered acceptable for library preparation and sequencing. Using Dynabeads Oligo (dT)25-61005 (Thermo Fisher, CA, USA), poly(A) RNA was isolated from total RNA in two cycles. Those compound doublets of DNA and RNA were converted into DNA duplexes. The second strand was digested with UDG enzyme, and then PCR was performed to create a library with a fragment size of 300bp ± 50bp. Finally, we carried out double-end sequencing using the Illumina NovaSeqTM 6000 (LC Bio-Technology CO., Ltd. Hangzhou, China).

### 2.5 Transcriptomic data processing and differential gene analysis

Forty samples of the murine small intestine split equally between normal and IIRI states, were processed to generate transcriptome data. Following RNA extraction using TRIzol, quality, and integrity assessments were conducted via NanoDrop and Bioanalyzer platforms. The mRNA libraries prepared using Dynabeads and sequenced on the Illumina NovaSeqTM 6000 system, were processed with Trimmomatic for quality trimming and STAR for alignment against the mouse Gencode M24 reference genome. Differential expression analysis was executed using the ‘limma’ package, applying a threshold of P-value <0.05 and an absolute log2 fold change >1.

### 2.6 Construction of co-expression networks

Sample clustering was performed using the hclust function in R with the average linkage method. Samples with a cut height of > 100 were considered outliers and removed from further analysis. The optimal soft-thresholding power for constructing a scale-free network was determined using the WGCNA package in R. Modules of co-expressed genes were identified, and the module most highly correlated with the IIRI phenotype was selected for further analysis.

### 2.7 Enrichment and interaction analyses

Candidate genes were pinpointed by intersecting differentially expressed genes and key module genes. GO and KEGG pathway enrichment analyses were performed using the ‘clusterProfiler’ package in R, with a p-value cutoff of 0.05 and a q-value cutoff of 0.1. The statistical significance of enrichment was determined using the hypergeometric test. A protein-protein interaction (PPI) network of these genes was visualized using STRING and further analyzed to identify central core genes using the MCODE algorithm in Cytoscape.

### 2.8 Application of machine learning for biomarker discovery

To refine the selection of potential biomarkers, machine learning techniques, specifically LASSO and SVM-RFE, were applied (20, 21). The intersection of the gene lists from both methods yielded a set of candidate biomarkers. ROC curves were generated using the pROC package in R. The area under the curve (AUC) was calculated to assess the diagnostic performance of the biomarkers. An AUC value of 0.5 indicates no discriminative power, while a value of 1.0 represents perfect discrimination.

### 2.9 Development of a predictive clinical model

A clinical nomogram incorporating the identified biomarkers was constructed using the ‘rms’ package to forecast the risk of IRI. The model’s predictive accuracy was assessed through calibration, decision curve analysis (DCA), and clinical impact curves, generated using the ggDCA package.

### 2.10 Immune feature and GSEA

The CIBERSORT algorithm was employed to quantify the infiltration of various immune cells in the tissue microenvironment. Relationships between these cells and the diagnostic genes were explored. Additionally, Gene Set Enrichment Analysis (GSEA) was performed to investigate relevant biological pathways and processes, utilizing mouse-specific gene sets.

### 2.11 IPA

An Ingenuity Pathway Analysis (IPA) (22) determined the activation states of pathways associated with the biomarkers. A threshold Z-score of ± 2 identified pathways significantly impacted by the biomarkers under study.

### 2.12 The analysis of the expression of biomarkers

For histological analysis (H&E staining and immunohistochemistry), we collected 2 cm segments from the standardized jejunal region (10 cm distal to the ligament of Treitz). These segments were immediately fixed in 4% paraformaldehyde. Multiple sections (5 μm thickness) were prepared from each sample, with analyses performed on sections taken at 0.5 cm intervals to ensure representative sampling. Expression levels of identified biomarkers were quantitatively validated using real-time PCR (qRT-PCR) on tissue samples from ten mice each in the sham and model groups. Total RNA was isolated using TRIZol reagent (Thermo Fisher, Shanghai, China, catalog no. 15596026) and reverse-transcribed to cDNA using the SureScript First-Strand cDNA Synthesis Kit (Servicebio, Wuhan, CN, catalog no. G3330). The qPCR reactions were facilitated by the PowerUp SYBR Green Master Mix (Thermo Fisher, Waltham, MA, USA, catalog no. A25742), employing specific primers listed in **Supplementary Table S1**. GAPDH served as the reference gene, and relative expression was calculated using the 2^−ΔΔCt^ method.

For immunohistochemical analysis, four tissue samples from each group were processed. Sections were blocked with 5% bovine serum albumin (BSA) in PBS for 1 hour at room temperature, then incubated overnight at 4°C with the following primary antibodies: anti-CCL7 (1:200, ABCAM, ab182793), anti-CD14 (1:100, ABCAM, ab221678), anti-CXCL1 (1:200, ABCAM, ab86436), anti-HMOX1 (1:150, ABCAM, ab13248), and anti-NFKBIA (1:100, ABCAM, ab32518). After washing three times with PBS containing 0.1% Tween-20 (PBST), sections were incubated with HRP-conjugated goat anti-rabbit IgG secondary antibody (1:500, Servicebio, catalog no. GB23303) for 1 hour at room temperature. The immunoreactivity was visualized using the DAB Substrate Kit (Vector Laboratories, Burlingame, CA, USA, catalog no. SK-4100). Images were captured using an Olympus BX53 light microscope equipped with a DP80 digital camera (Olympus, Tokyo, Japan) at 200x magnification, and analyzed quantitatively using ImageJ-pro-plus software (version 6.0, NIH, USA). Statistical analysis was performed using Prism software (GraphPad Software, San Diego, CA, USA), ensuring that data interpretation adhered to rigorous standards.

### 2.13 Isolation of exosomes from bovine milk

Exosomes were isolated from commercially available bovine milk (23). The milk underwent sequential centrifugation steps: initial centrifugation at 5000 × g for 30 minutes to remove fat and debris, followed by a second spin at 12,000 × g for 1 hour at 4°C (Avanti J-E Centrifuge, Beckman Coulter, Brea, USA). The supernatant was filtered through a 40-μm cell strainer (SPL Life Sciences, Pocheon-si, Republic of Korea, catalog no. 93040) and subjected to ultracentrifugation at 35,000 × g for 1 hour and then at 70,000 × g for 3 hours at 4°C (Optima XE-100, Beckman Coulter). The middle layer containing exosomes was collected, and passed through syringe filters of decreasing pore sizes (0.8, 0.45, and 0.2 μm, Sartorius, Göttingen, Germany; catalog nos. 16592-K, 16555-K, 16534-K), and centrifuged at 100,000 × g for 1 hour to pellet the exosomes. The purified exosomes were resuspended in phosphate-buffered saline (PBS, GenDEPOT, Katy, USA, catalog no. CA008-050) and stored at 4°C overnight to ensure homogeneity.

### 2.14 Electroporation of M-Exo and siRNA loading

For electroporation, RNase-free 10 × PBS (AM9625, Invitrogen, Waltham, USA) was diluted 1/50 in diethylpyrocarbonate (DEPC)-treated water (WR2004-050-00, Biosesang, Seongnam-si, Republic of Korea) and used as the electroporation buffer. Exosome particles were quantified using nanoparticle tracking analysis (NTA) with a NanoSight NS300 system (Malvern Panalytical, UK). Three independent measurements were performed for each sample to ensure accuracy. M-Exos were then added to the electroporation buffer to prepare a solution with 1.6 × 1011 particles/mL.

All electroporation experiments were performed using Gene Pulser Xcell Electroporation Systems (1652660, BIO-RAD, Hercules, USA) and Gene Pulser/MicroPulser Electroporation Cuvettes with a 0.4 cm gap (1652088, BIO-RAD, Hercules, USA) according to the electroporation conditions.

The siRNA targeting CCL7 (siCCL7) was purchased from Guangzhou RiboBio Co., Ltd. (Ribobio, Guangzhou, China) with the product number siG170420120148-1-5 and a standard specification of 5 nmol. The sense and antisense sequences of siCCL7 were as follows: Sense: 5’-GCAAGAAAGCUUAAGGAAU dTdT-3’, Antisense: 3’- dTdT CGUUCUUUCGAAUUCCUUA-5’. After electroporation, siCCL7 was added to the M-Exos at a final concentration of 100 nM to prepare M-Exo/siCCL7. The M-Exo/siCCL7 complex was then incubated at 4°C for 3 h with mild shaking to facilitate restoration. To remove unloaded free siRNA, centrifugation was performed at 12,000 × g for 10 min using a 30 kDa-Amicon Ultra-0.5 centrifugal filter unit (UFC503096, Merck Millipore, Burlington, USA). The final M-Exo/siCCL7 preparation was adjusted to a concentration of 1×1012 particles/mL in PBS, and 100 μL was administered to each mouse by oral gavage (corresponding to a dose of 1×1011 particles per mouse), once daily for 2 consecutive days before the induction of IIRI.

### 2.15 Transmission electron microscopy analysis of exosomes

The morphology and size of exosomes were observed by TEM as previously described (24). Briefly, 10 μL of exosome suspension was placed on a copper grid and allowed to absorb for 5 min. The excess liquid was removed with filter paper, and the sample was negatively stained with 10 μL of 20 mL/L uranyl acetate solution for 1 min. The excess liquid was removed again, and the copper grid was dried at room temperature. The morphology and size of the exosomes were observed and photographed using a transmission electron microscope (H-7650, Hitachi, Tokyo, Japan).

### 2.16 Enzyme-linked immunosorbent assay for serum CCL7

Blood samples were collected via cardiac puncture immediately after euthanasia. Blood was allowed to clot at room temperature for 30 minutes, then centrifuged at 3,000 × g for 15 minutes at 4°C to obtain serum. The serum was carefully collected, aliquoted, and stored at -80°C until analysis. Serum levels of CCL7 were measured using a mouse CCL7 ELISA kit (MCC070, R&D Systems, Minneapolis, MN, USA) according to the manufacturer’s instructions. Serum levels of CCL7 were measured using a mouse CCL7 ELISA kit (MCC070, R&D Systems, Minneapolis, MN, USA) according to the manufacturer’s instructions. Briefly, serum samples were diluted with sample dilution buffer and added to the ELISA plate pre-coated with capture antibody. After incubation and washing, the detection antibody was added, followed by the addition of substrate solution. The optical density (OD) was measured at 450 nm using a microplate reader (Bio-Rad, Hercules, CA, USA). The concentration of CCL7 was calculated based on the standard curve.

### 2.17 Histological assessment and Chiu’s score

Intestinal tissues were fixed in 40 mL/L paraformaldehyde, embedded in paraffin, and sectioned at 5 μm thickness. The sections were stained with hematoxylin and eosin (H&E) for histological assessment. The severity of intestinal injury was evaluated using Chiu’s score (25) based on the following criteria: 0, normal mucosa; 1, development of subepithelial space at the tip of the villus; 2, extension of the subepithelial space with moderate lifting of the epithelial layer; 3, massive epithelial lifting with a few denuded villi; 4, denuded villi with exposed capillaries; and 5, disintegration of the lamina propria, ulceration, and hemorrhage. The scores were assessed by two independent pathologists who were blinded to the experimental groups.

### 2.18 Measurement of serum diamine oxidase activity

Serum diamine oxidase (DAO) activity was measured using a commercial kit (A088-1-1, Nanjing Jiancheng Bioengineering Institute, Nanjing, China) according to the manufacturer’s protocol. Briefly, serum samples were incubated with the reaction mixture containing cadaverine dihydrochloride as the substrate. The enzymatic reaction was terminated, and the absorbance was measured at 436 nm using a microplate reader. DAO activity was calculated based on the standard curve and expressed as U/L.

### 2.19 Measurement of serum lactate dehydrogenase levels

Serum lactate dehydrogenase (LDH) levels were determined using a commercial LDH assay kit (A020-2-2, Nanjing Jiancheng Bioengineering Institute, Nanjing, China) following the manufacturer’s instructions. Briefly, serum samples were incubated with the reaction mixture, and the absorbance was measured at 450 nm using a microplate reader. LDH levels were calculated based on the standard curve and expressed as U/L.

### 2.20 Statistical analysis

Data normality was first assessed using the Shapiro-Wilk test. For normally distributed data, a two-tailed Student’s t-test was used to confirm the statistical significance of differences between two groups, and a one-way analysis of variance (ANOVA) with the Tukey-Kramer *post hoc* test was used for multi-group comparisons. For non-parametric data (Chiu’s scores and relative expression data), the Mann-Whitney U test was used for two-group comparisons, and the Kruskal-Wallis test followed by Dunn’s *post-hoc* test was used for multiple-group comparisons. Results are presented as mean ± standard deviation (SD) for parametric data and median with interquartile range for non-parametric data. Statistical analysis was performed using Prism software (version 9.0). Statistical significance was set as follows: not significant (ns) > 0.05, *p < 0.05, **p < 0.01, ***p < 0.001, and ****p < 0.0001.

## 3 Results

### 3.1 Identification and functional analysis of candidate genes in IRI

**Figure 2:**
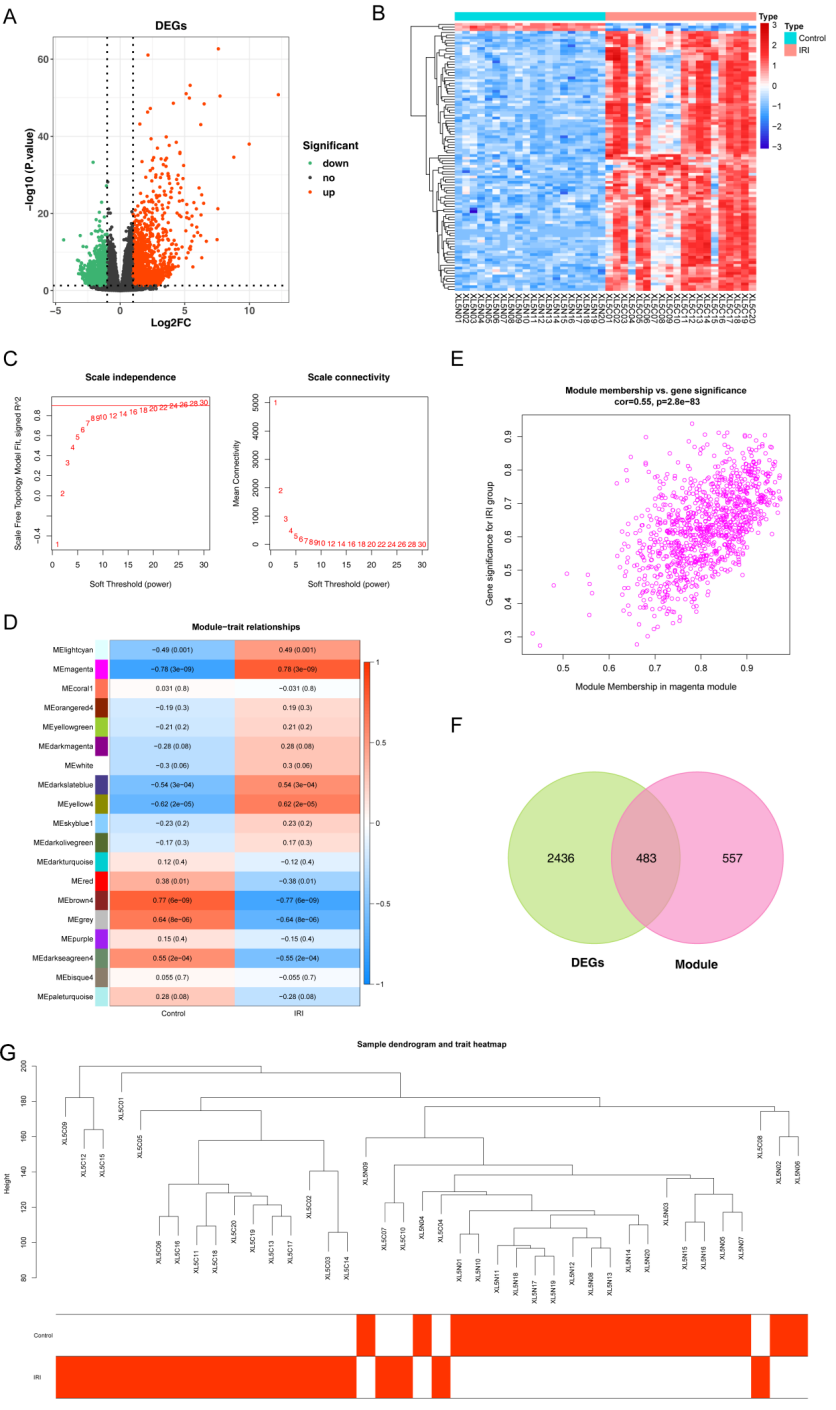
Identification of candidate genes associated with IIRI. **(A, B)** Heatmap and volcano plot depicting the 2,919 differentially expressed genes (DEGs) between the IIRI and normal groups. **(C)** Analysis of the scale-free fit index (left) and mean connectivity (right) for selecting the optimal soft-thresholding power in WGCNA. **(D)** GO pathway analysis of the IIRI vs. normal groups. **(E)** Scatter plot of the key module correlated with the IIRI vs. normal groups. **(F)** Venn diagram illustrating the overlap between IIRI-related genes and DEGs. **(G)** Sample dendrogram and trait heatmap.

The base quality Q30 of each sample was above 90% (**Supplementary Table S2**). Using the mouse reference genome alignment, the mapping rate of all samples was above 75%, suggesting that the sequencing quality was very good (**Supplementary Table S3**). 2919 DEGs were identified in the IRI vs. normal group, including 1301 down-regulated and 1618 up-regulated genes (**Figures 2A, B**). To identify key modules associated with IRI vs. normal groups, we conducted a WGCNA. There were no outlier samples (**Figure 2**). The optimal soft threshold was 28. When the mean connectivity tended to 0, the ordinate scale-free fit index signed R^2 approached the threshold value of 0.9 (red line) (**Figure 2C**). A total of 19 modules were obtained (**Figure 2D**). The MEmagenta module showed a significant correlation with IRI in comparison to the normal groups (**Figure 2E**). Thus, 1040 key module genes related to IRI vs. normal groups were obtained. Furthermore, 483 candidate genes were identified by overlapping key module genes that were related to IRI vs. normal groups and DEGs (**Figure 2F**).

**Figure 3:**
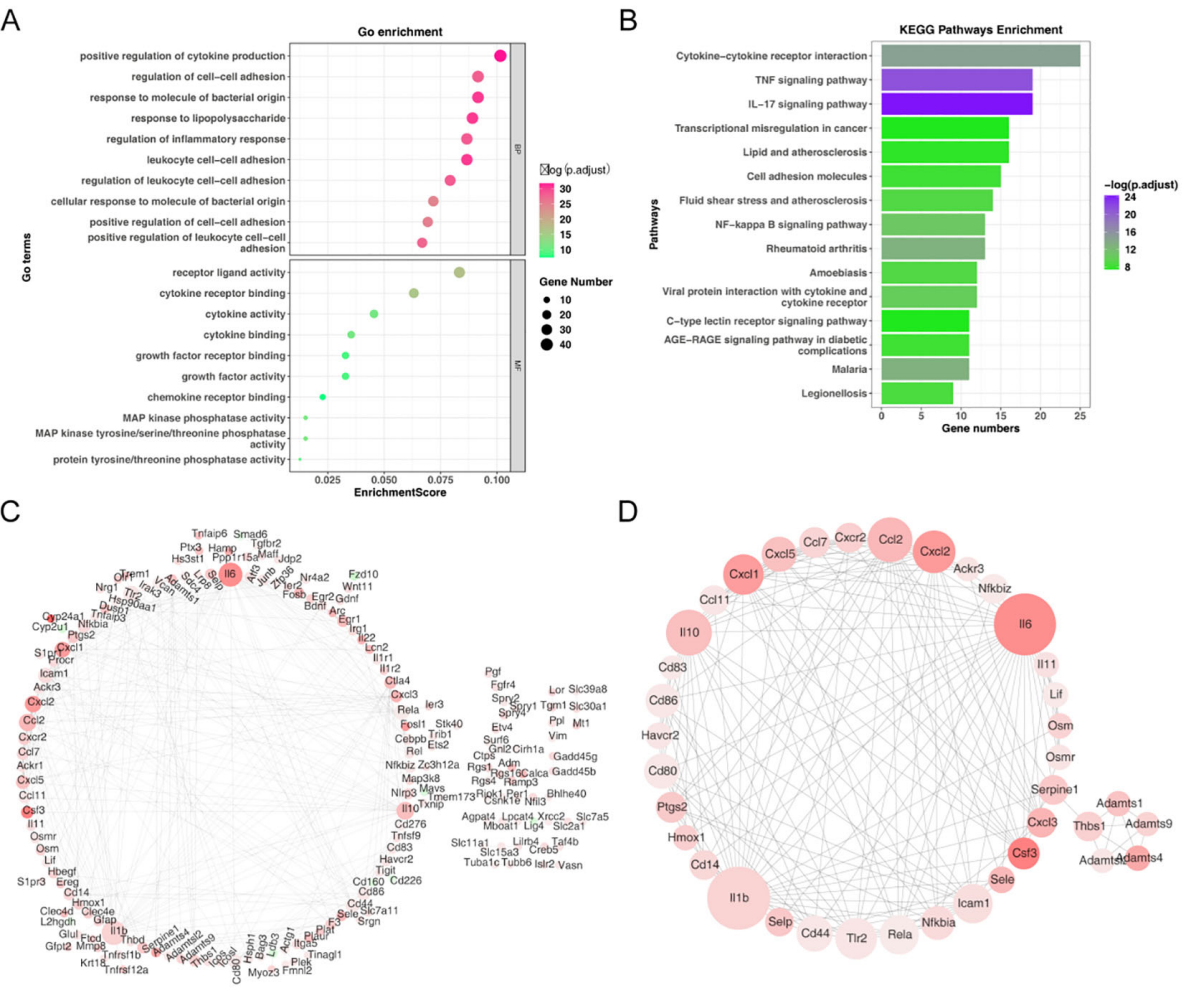
Functional enrichment analysis of candidate genes. **(A)** GO enrichment bubble plot of candidate genes. **(B)** KEGG pathway enrichment analysis of candidate genes. **(C)** Protein-protein interaction (PPI) network of candidate genes. **(D)** Identification of 38 core genes using the Cytoscape MCODE plug-in.

To further investigate the potential function of candidate genes, we conducted a functional enrichment analysis. These candidate genes were principally involved in the ‘positive regulation of cytokine production’ process (**Figure 3A**). Additionally, the KEGG analysis demonstrated that these candidate genes were mainly relevant to the ‘NF-kappa-B signaling pathway’ (**Figure 3B**). Furthermore, the PPI network of candidate genes was constructed, consisting of 176 nodes and 394 edges (**Figure 3C**). 38 core genes were identified by the Cytoscape plug-in according to MCODE degree. The network of core genes had 38 nodes and 183 edges, including Cxcl1, Cxcl5, Ccl7, Cxcr2, Ccl2, and Cxcl2, et al. (**Figure 3D**).

### 3.2 Screening and clinical predictive analysis for IRI patients

**Figure 4:**
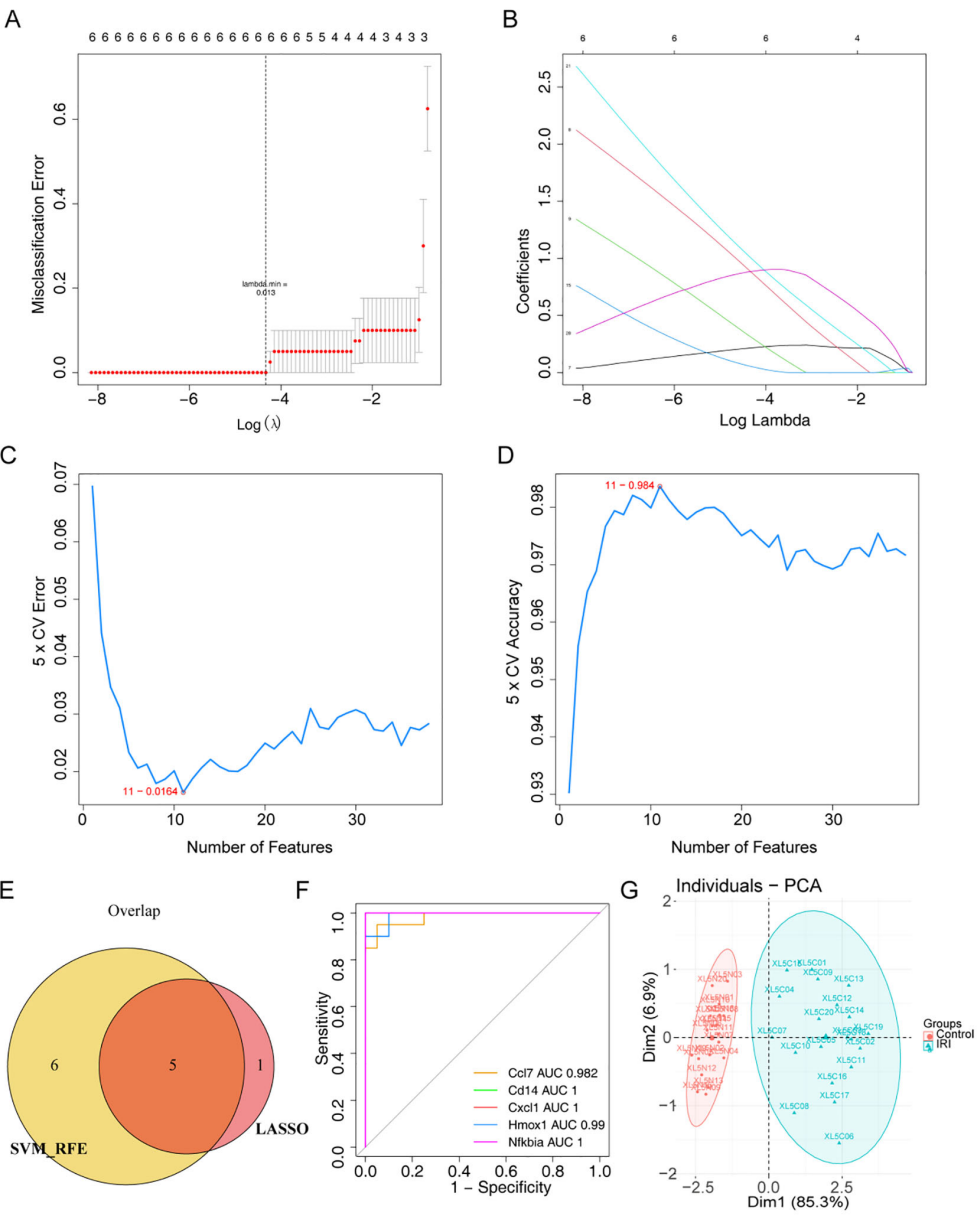
Screening of biomarkers in IIRI. **(A, B)** LASSO coefficient profiles and 6-fold cross-validation for optimal tuning parameter (λ) selection using LASSO. **(C, D)** Estimation of 5-fold cross-validation error and accuracy using support vector machine recursive feature elimination (SVM-RFE). **(E)** Venn diagram depicting the overlap of 5 genes identified by LASSO and SVM-RFE. **(F)** ROC curves for Ccl7, Cd14, Cxcl1, Hmox1, and Nfkbia. **(G)** PCA plot of the biomarkers distinguishing normal and IIRI samples.

To further identify the key genes, Lasso regression analysis was performed on 38 core genes to uncover the optimal ones. Ultimately, 6 feature genes were obtained, including Ccl2, Ccl7, Cd14, Cxcl1, Hmox1, and Nfkbia (**Figures 4A, B**). Meanwhile, the SVM-RFE algorithm retained 11 feature genes, namely Cd14, Adamts1, Cxcl1, Hmox1, Il10, Csf3, Adamts4, Ccl7, Ccl2, Thbs1, and Nfkbia (**Figures 4C, D**). Subsequently, we obtained 5 overlapping genes, including Ccl7, Cd14, Cxcl1, Hmox1, and Nfkbia (**Figure 4E**). We defined them as biomarkers in IRI. The AUC values were greater than 0.9, indicating that the biomarkers exhibited excellent diagnostic accuracy (**Figure 4F**). PCA analysis showed that biomarkers could effectively distinguish normal samples from IRI samples (**Figure 4G**).

**Figure 5:**
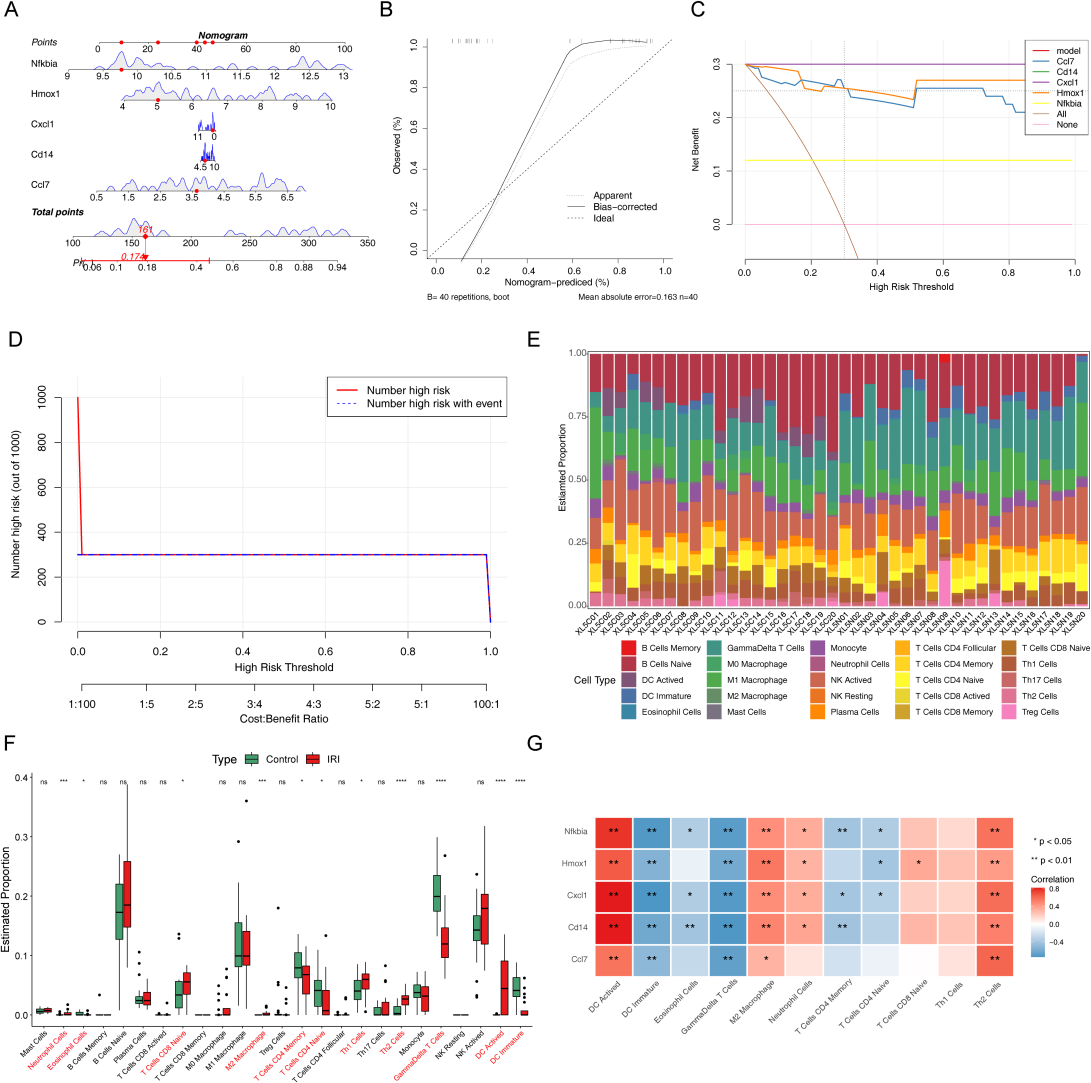
Clinical predictive analysis for IIRI patients. **(A)** Nomogram based on the risk score and containing biomarkers. **(B)** Calibration curve of the nomogram for prediction. **(C)** Decision curve analysis (DCA) curve containing biomarkers. **(D)** Clinical impact curve containing biomarkers. **(E)** Enrichment analysis of the immune microenvironment in IIRI using 25 immune gene sets. **(F)** Box plots depicting differences in immune cell infiltration between the two groups. **(G)** Heatmap of the correlation between biomarkers and differentially infiltrated immune cells. *p < 0.05, **p < 0.01, ***p < 0.001, ****p < 0.0001.

To evaluate the diagnostic ability of biomarkers, a nomogram containing the biomarkers was generated (**Figure 5A**). The calibration, DCA, and clinical impact curves demonstrated the effectiveness of the diagnostic model’s performance (**Figures 5B-D**).

### 3.3 Immune infiltration and functional analysis

To investigate the immune microenvironment of IRI, we examined the abundance of 25 immune gene sets in two sample groups (**Figure 5E**). Notably, there were significant differences in the abundances of 11 immune cell types, including neutrophils, eosinophils, CD8 naive T cells, M2 macrophages, CD4 naive T cells, CD4 memory T cells, Th2 cells, Th1 cells, γδ T cells, activated dendritic cells (DC), and immature DC (**Figure 5F**). The correlation between biomarkers and these differential immune cells is illustrated in **Figure 5G**. Five biomarkers showed significant positive correlations with activated DC, M2 macrophages, and Th2 cells, while all biomarkers were negatively correlated with immature DC and γδ T cells.

### 3.4 IPA analysis

**Figure 6:**
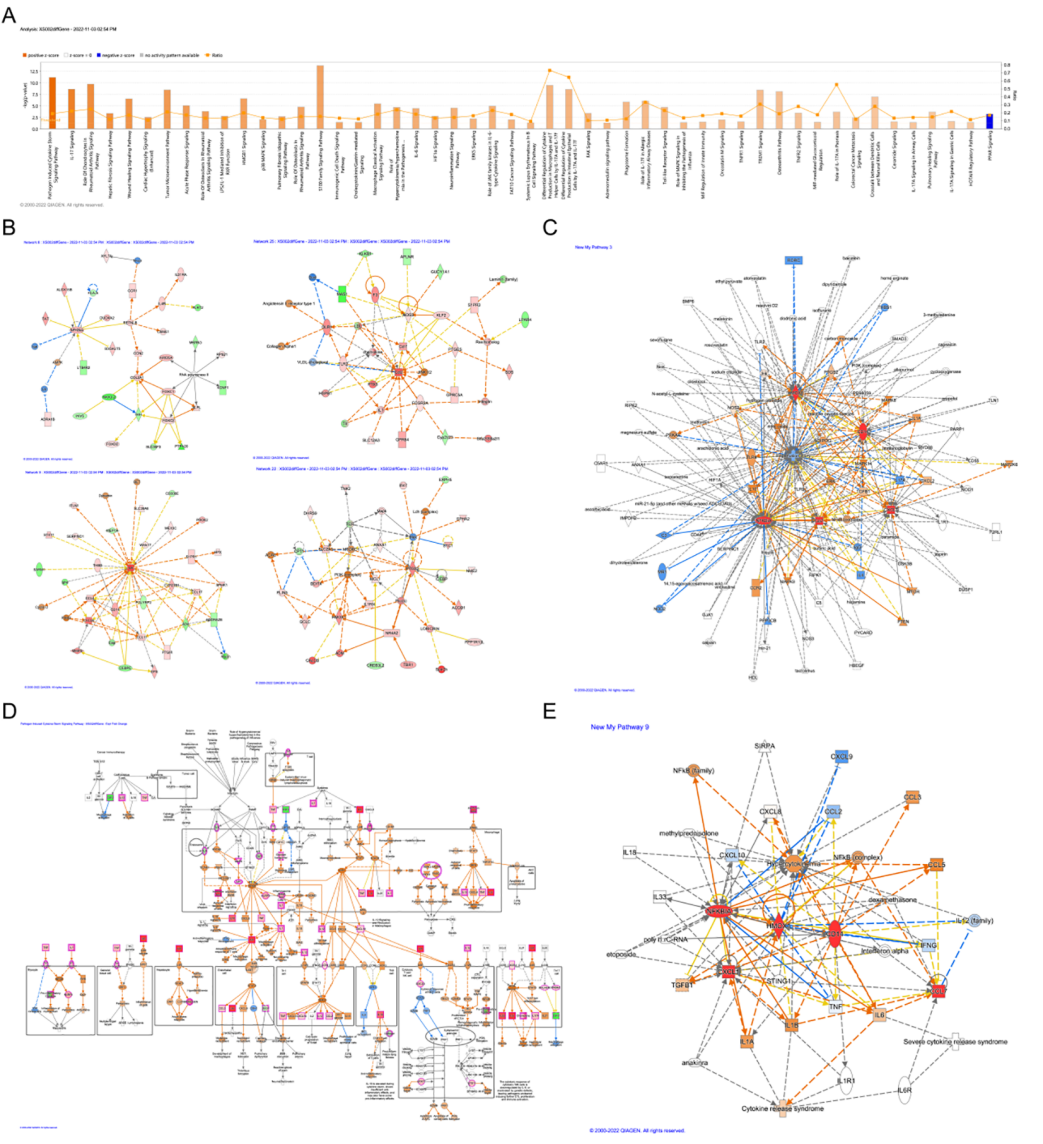
IPA analysis. **(A)** Significant classical pathway enrichment results. **(B)** Molecular interaction network of Ccl7, Cd14, Hmox1, and Nfkbia. **(C)** Regulatory relationship between biomarkers and reperfusion injury. **(D)** Regulatory relationship of the ‘Pathogen Induced Cytokine Storm Signaling’ pathway (highest Z-score). **(E)** Interaction network between biomarkers and cytokines.

Classical pathway analysis using IPA indicated that all differentially expressed genes (DEGs) were associated with 50 pathways. Only the ‘PPAR Signaling’ pathway was inhibited (**Figure 6A**). The molecular interaction network of Ccl7, Cd14, Hmox1, and Nfkbia is presented in **Figure 6B**. We observed that Cd14 was associated with THBD. In the activated state of biomarkers, we demonstrated the regulatory relationship between biomarkers and reperfusion injury (**Figure 6C**). The regulatory relationship of ‘Pathogen Induced Cytokine Storm Signaling’ had the highest Z-score (**Figure 6D**). Among these interactions, biomarkers might interact with several cytokines, such as IFNG, IL6, and L1A (**Figure 6E**).

### 3.5 Validation of biomarker expression

**Figure 7:**
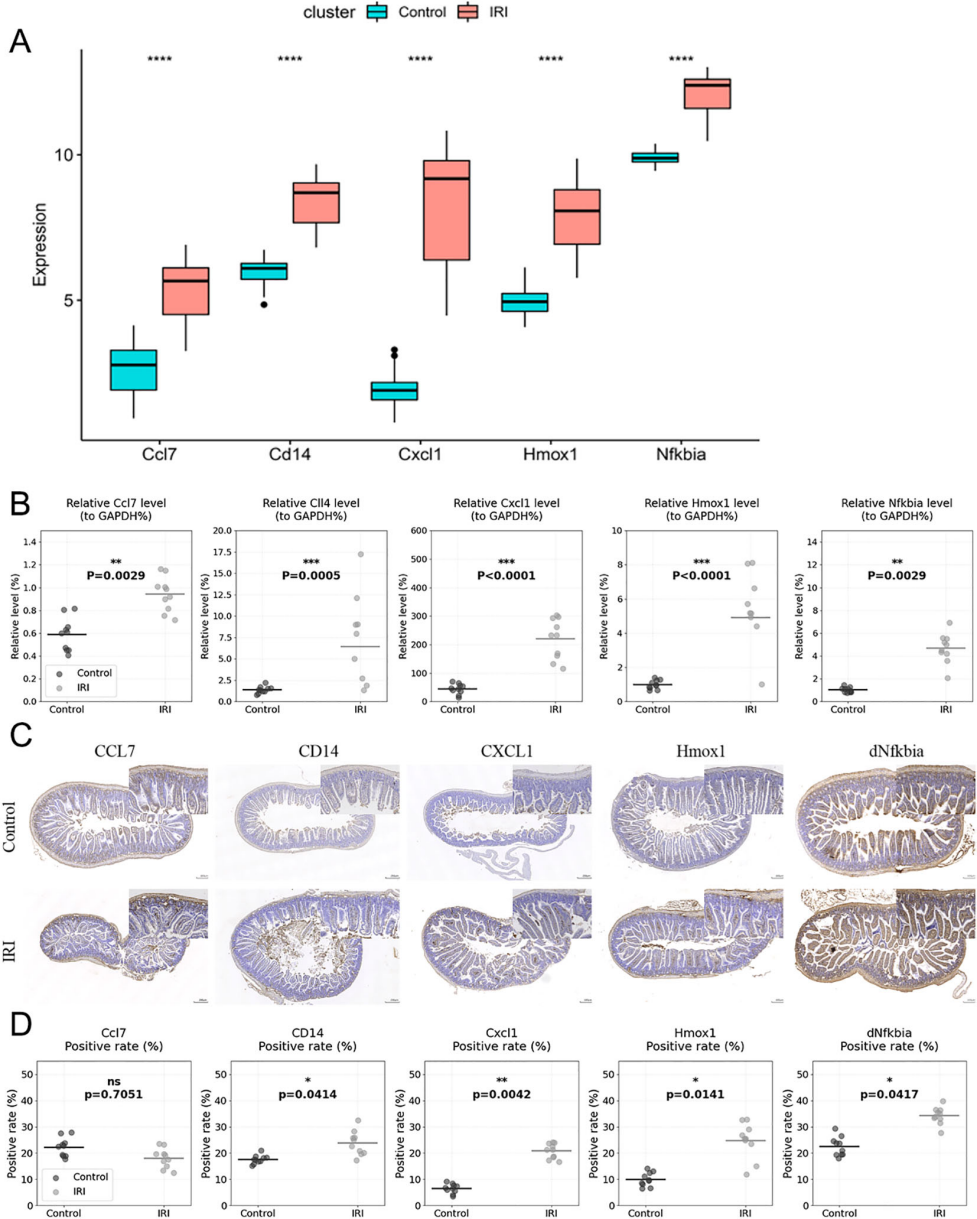
Validation of biomarker expression. **(A)** Schematic representation of biomarker expression validation in mouse tissue samples by RT-qPCR. **(B)** Scatter plots showing individual data points of qRT-PCR validation for Ccl7, Cd14, Cxcl1, Hmox1, and Nfkbia expression in IIRI (n=10) vs. control samples (n=10), with median and interquartile range. **(C, D)** Quantification of immunohistochemical staining shown as scatter plots with individual values (n=4 per group) for Ccl7, Cd14, Cxcl1, Hmox1, and Nfkbia expression in IIRI vs. control samples, with mean ± SD. *p < 0.05, **p < 0.01, ***p < 0.001, ****p < 0.0001 vs. control.

As illustrated in **Figure 7A**, we observed higher expression levels of Ccl7, Cd14, Cxcl1, Hmox1, and Nfkbia in the IIRI group based on the sequencing data. We validated the expression in mouse tissue samples using RT-qPCR. RT-qPCR analysis revealed significant upregulation of the candidate biomarkers in IIRI samples compared to controls. The relative expression levels were: Cd14 [median 3.8-fold (IQR: 3.2-4.3), p < 0.01], Cxcl1 [median 4.2-fold (IQR: 3.7-4.8), p < 0.01], Hmox1 [median 2.9-fold (IQR: 2.5-3.4), p < 0.01], and Nfkbia [median 3.1-fold (IQR: 2.7-3.6), p < 0.01] compared to control samples. Ccl7 showed variation in expression [median 0.8-fold (IQR: 0.6-1.1), p > 0.05] (**Figure 7B**). We further confirmed the expression in mouse tissue samples by immunohistochemistry. Consistent with the sequencing results, the expression levels of Cd14, Cxcl1, Hmox1, and Nfkbia were markedly higher in the IRI group compared to control samples (**Figures 7C, D**). While individual validation experiments showed variable CCL7 expression, our integrated bioinformatics analysis consistently identified CCL7 as a hub gene in the protein-protein interaction network. Furthermore, previous literature has established CCL7’s role in inflammatory responses. The variability in validation results may reflect the dynamic nature of the inflammatory response and temporal differences in gene expression. We selected CCL7 for therapeutic targeting based on its central position in our network analysis and its known functions in inflammatory cascades.

### 3.6 M-Exo/siCCL7 treatment attenuates intestinal ischemia-reperfusion injury

**Figure 8:**
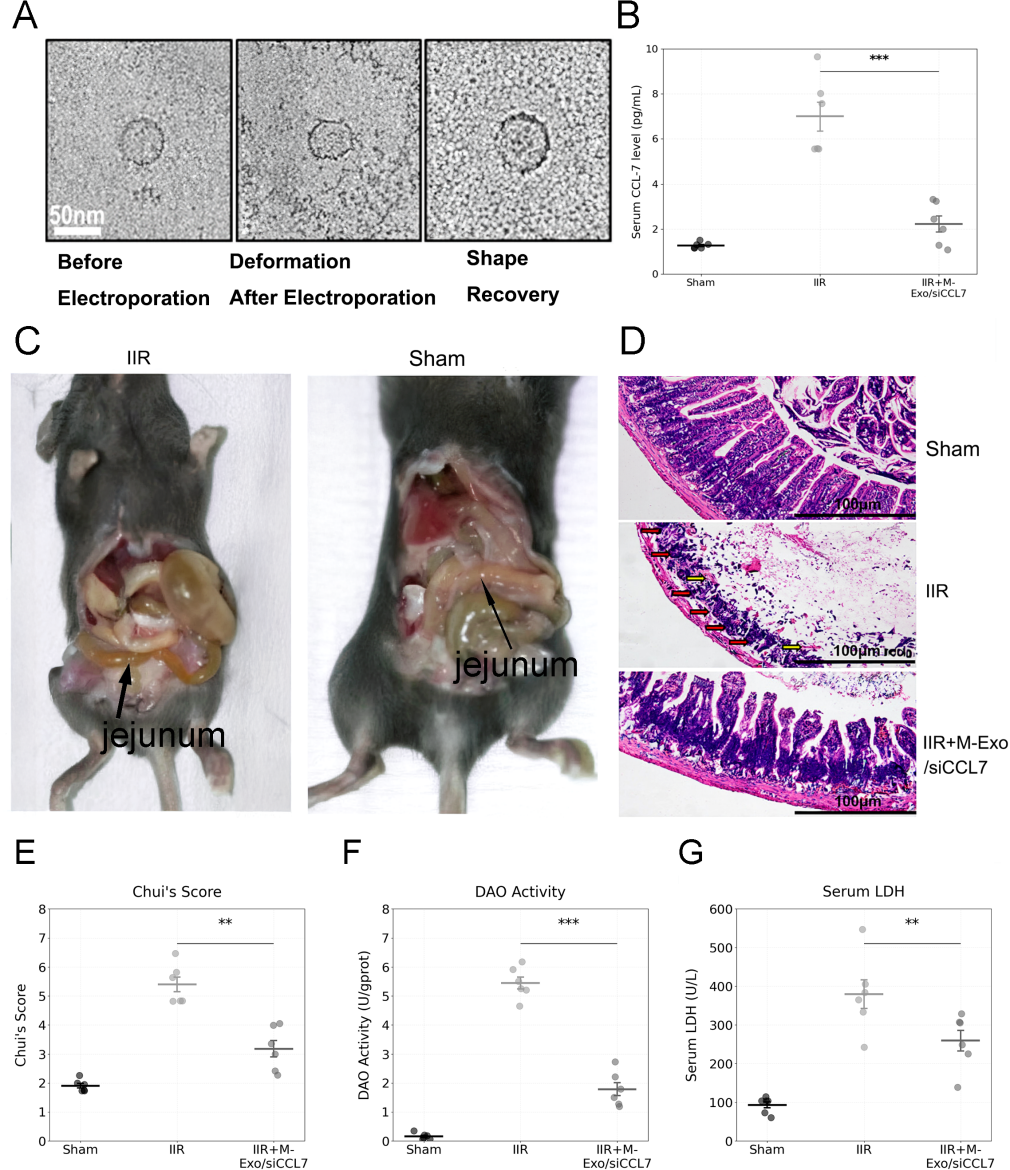
M-Exo/siCCL7 treatment attenuates intestinal ischemia-reperfusion injury. **(A)** Transmission electron microscopy images depicting the morphology of milk-derived exosomes (M-Exos) before and after electroporation. **(B)** Serum CCL7 levels in the sham, IIRI, and M-Exo/siCCL7-treated (1×10^11^ particles/mouse, once daily for 2 days) groups. **(C)** Representative gross anatomical photographs of jejunum before (normal pink coloration) and after (dark red/purple with visible edema) ischemia-reperfusion injury, showing macroscopic changes in intestinal appearance. **(D)** Representative histological images (H&E staining) of intestinal tissues from the three groups. **(E)** Chiu’s score for the assessment of intestinal injury severity. **(F)** Serum diamine oxidase activity as a marker of intestinal barrier function. **(G)** Serum lactate dehydrogenase levels as an indicator of tissue damage. Data are presented as mean ± SD; **p < 0.01, ***p < 0.001.

Our findings from the initial transcriptome analysis and bioinformatics studies identified CCL7 as a key hub gene in the protein-protein interaction network (as shown in **Figure 3D**). Drawing inspiration from other studies, our research team explored the use of milk-derived exosomes (M-Exos) for siRNA delivery and evaluated their therapeutic effects in an IIRI mouse model. Transmission electron microscopy confirmed the morphological changes and recovery of M-Exos after electroporation (**Figure 8A**). M-Exo/siCCL7 treatment significantly downregulated serum CCL7 levels compared to the IIR group (**Figure 8B**), validating the effective modulation of CCL7 expression.The ischemia-reperfusion injury predominantly manifested in the jejunum, with the most severe and consistent changes observed in the segment 10 cm distal to the ligament of Treitz. This region exhibited characteristic features including Visible edema with intestinal wall thickening; Color changes from normal pink to dark red/purple, indicating congestion; Clear demarcation between affected and normal segments; and Reduced peristalsis in the affected segments. The injury pattern was consistent across all animals in the IIRI group, while the intestines of sham-operated animals maintained a normal appearance(**Figure 8C**). Histological assessment using Chiu’s scoring system revealed significant tissue damage in the IIRI group [median score 4.0 (IQR: 3.5-4.5)] compared to the sham group [median score 0.0 (IQR: 0.0-0.5), p < 0.001]. M-Exo/siCCL7 treatment significantly reduced the injury severity [median score 2.0 (IQR: 1.5-2.5), p < 0.01 compared to the IIRI group]. Histological analysis revealed that M-Exo/siCCL7 intervention alleviated inflammatory cell infiltration, mucosal ulceration, and crypt loss in the intestine (**Figures 8D, E**). Serum CCL7 levels showed a similar trend, with significant elevation in the IIRI group [median 185.3 pg/mL (IQR: 165.2-205.4)] compared to sham [median 42.1 pg/mL (IQR: 35.4-48.9), p < 0.001], while M-Exo/siCCL7 treatment effectively reduced these levels [median 95.6 pg/mL (IQR: 85.3-105.9), p < 0.01 compared to IIRI group]. Decreased serum diamine oxidase activity (**Figure 8F**), and attenuated serum LDH levels (**Figure 8G**) compared to the IIR group, indicating protection of the intestinal mucosa, barrier function, and reduced tissue damage. While our histological findings and serum DAO levels suggest improved tissue integrity, direct measurement of barrier function through permeability assays would provide more definitive evidence. Nevertheless, our current data indicates that targeting CCL7 using M-Exo-mediated siRNA delivery shows therapeutic potential in IIRI treatment.

## 4 Discussion

Intestinal ischemia-reperfusion injury (IIRI) is a severe clinical condition associated with high morbidity and mortality. Despite advances in understanding the pathophysiology of IIRI, effective diagnostic and therapeutic strategies remain limited. In this study, we employed a transcriptome sequencing approach to identify key biomarkers and potential therapeutic targets in a mouse model of IIRI. Our analysis revealed five differentially expressed genes (Ccl7, Cd14, Cxcl1, Hmox1, and Nfkbia) that were significantly upregulated in the IIRI group compared to the sham group. These biomarkers were found to be enriched in inflammatory pathways, underscoring the central role of inflammation in the pathogenesis of IIRI.

IIRI is characterized by a complex inflammatory cascade involving the activation of innate immune cells, the release of pro-inflammatory cytokines and chemokines, and the infiltration of leukocytes into the intestinal tissue (3, 4, 26, 27). This excessive and uncontrolled inflammation leads to tissue damage, mucosal barrier dysfunction, and systemic complications (28, 29). The biomarkers identified in our study are known to play critical roles in various aspects of the inflammatory response. Cd14 is a pattern recognition receptor that mediates the activation of toll-like receptor 4 (TLR4) signaling in response to lipopolysaccharide (LPS) (30–32). Cxcl1 is a potent neutrophil chemoattractant that promotes neutrophil infiltration and activation (33–35). Hmox1 is an enzyme with anti-inflammatory and antioxidant properties that helps to counteract oxidative stress and inflammation (36, 37). Nfkbia is a key regulator of the NF-κB signaling pathway, which is a central mediator of inflammatory gene expression (38–40). The upregulation of these biomarkers in the IIRI group suggests their involvement in the initiation and amplification of the inflammatory response during IIRI.

Among the identified biomarkers, Ccl7 emerged as a hub gene with broad implications in inflammatory diseases and cancer. Ccl7, also known as monocyte chemoattractant protein-3 (MCP-3), is a chemokine that attracts monocytes, lymphocytes, and eosinophils to sites of inflammation (41–43). Elevated Ccl7 expression has been reported in various inflammatory conditions, including inflammatory bowel disease, rheumatoid arthritis, and multiple sclerosis (44). In the context of IIRI, our findings suggest that Ccl7 may contribute to the recruitment of inflammatory cells and exacerbation of tissue damage. Interestingly, Ccl7 has also been implicated in the pathogenesis of several types of cancer, such as colorectal cancer, breast cancer, and lung cancer (45, 46). In these malignancies, Ccl7 has been associated with tumor progression, metastasis, and poor prognosis. The overexpression of Ccl7 in cancer is thought to promote the recruitment of tumor-associated macrophages and other immune cells that create a pro-tumorigenic microenvironment (47). These findings highlight the diverse roles of Ccl7 in regulating inflammatory responses and cell migration across different pathological conditions.

Given the broad involvement of Ccl7 in inflammatory diseases and cancer, targeting Ccl7 could have significant therapeutic potential. In our study, we explored the therapeutic efficacy of milk-derived exosomes (M-Exos) loaded with siRNA targeting Ccl7 (M-Exo/siCCL7) in a mouse model of IIRI. Exosomes are natural nanoparticles that have emerged as promising drug delivery vehicles due to their biocompatibility, stability, and ability to cross biological barriers (48, 49). M-Exos, in particular, have been shown to possess intrinsic anti-inflammatory and immunomodulatory properties, making them attractive candidates for the treatment of inflammatory diseases (49). Moreover, exosomes can efficiently encapsulate and deliver siRNAs to target cells, overcoming the challenges of siRNA instability and poor cellular uptake (50). Our results demonstrated that M-Exo/siCCL7 treatment effectively attenuated intestinal inflammation and injury in IIRI mice, as evidenced by reduced histological damage, decreased serum markers of intestinal barrier dysfunction, and attenuated systemic inflammation. These findings suggest that M-Exo-mediated delivery of siRNA targeting Ccl7 could be a promising therapeutic approach for IIRI.

The use of exosome-based siRNA delivery has several advantages over conventional siRNA delivery methods. Exosomes are natural carriers that can protect siRNAs from degradation and facilitate their uptake by target cells (51). The lipid bilayer of exosomes can fuse with the cell membrane, allowing for efficient delivery of the siRNA cargo into the cytoplasm (52). Exosomes also have the ability to cross biological barriers, such as the blood-brain barrier, which expands their potential therapeutic applications (53). Furthermore, exosomes can be engineered to display specific ligands or receptors on their surface, enabling targeted delivery to specific cell types or tissues (54). This targeted delivery approach can enhance the specificity and efficacy of siRNA-based therapies while minimizing off-target effects.

While our study provides proof-of-concept for the therapeutic potential of M-Exo/siCCL7 in IIRI, there are several limitations and future directions to be considered. First, we focused on a single time point after IIRI, which may not fully capture the dynamic changes in gene expression and inflammatory responses during the course of the disease. Second, while we observed improvements in histological parameters and serum markers, our study did not include direct measurements of intestinal barrier function through permeability assays, which would provide more definitive evidence of barrier protection. Future studies should incorporate such measurements to comprehensively evaluate the protective effects of M-Exo/siCCL7 treatment on intestinal barrier function. Third, although we demonstrated the therapeutic efficacy of M-Exo/siCCL7 in a mouse model, translation to clinical application will require extensive safety and efficacy studies in larger animal models and humans. The long-term safety, immunogenicity, and potential side effects of M-Exo-based therapies need to be carefully evaluated. Moreover, the optimal dosing, route of administration, and treatment regimen for M-Exo/siCCL7 need to be determined in future studies.

## Data Availability

The datasets presented in this study can be found in online repositories. The names of the repository/repositories and accession number(s) can be found below: PRJNA1040153 (SRA).
