# Supplementary material for: Inflammatory biomarkers and therapeutic potential of milk exosome-mediated CCL7 siRNA in murine intestinal ischemia-reperfusion injury
Source: Front Immunol. 2025 Jan 20;15:1513196. doi: 10.3389/fimmu.2024.1513196 (PMC11788141; doi:10.3389/fimmu.2024.1513196)
Supplement: Supplementary file 1 [file Table1.docx]

**Table S1** The primer sequences for PCR

| Primer | Sequence 5′–3′ | |
| --- | --- | --- |
| Ccl7 F | GGATCTCTGCCACGCTTCTG | |
| Ccl7 R | CCTCCTCGACCCACTTCTGA | |
| Cd14 F | ACTGAAGCCTTTCTCGGAGC |  |
| Cd14 R | TGAAAGCGCTGGACCAATCT |  |
| Cxcl1 F | ACTCAAGAATGGTCGCGAGG |  |
| Cxcl1 R | GTGCCATCAGAGCAGTCTGT |  |
| Hmox1 F | GAAATCATCCCTTGCACGCC |  |
| Hmox1 R | CCTGAGAGGTCACCCAGGTA |  |
| Nfkbia F | AGGACGAGGAGTACGAGCAA |  |
| Nfkbia R | CGTGGATGATTGCCAAGTGC |  |
| internal reference-M-Gapdh F | CCTTCCGTGTTCCTACCCC |  |
| internal reference-M-Gapdh R | GCCCAAGATGCCCTTCAGT |  |

**Table S2** The base quality Q30 of each sample

| **Sample** | **Raw Reads** | **Clean Reads** | **Raw Base(G)** | **Clean Base(G)** | **Effective Rate (%)** | **Q20** | **Q30** | **GC Content(%)** | **Group** |
| --- | --- | --- | --- | --- | --- | --- | --- | --- | --- |
| XL5C01 | 38449030 | 28149608 | 5.77 | 4.17 | 73.2128 | 4.11(98.51%) | 3.96(94.99%) | 47.69 | Module |
| XL5C02 | 39296462 | 31171986 | 5.89 | 4.63 | 79.3252 | 4.57(98.59%) | 4.41(95.17%) | 48.39 | Module |
| XL5C03 | 40965694 | 34178590 | 6.14 | 5.08 | 83.4322 | 5.01(98.62%) | 4.84(95.26%) | 48.6 | Module |
| XL5C04 | 43969430 | 38599882 | 6.6 | 5.74 | 87.788 | 5.66(98.60%) | 5.46(95.18%) | 48.45 | Module |
| XL5C05 | 36484828 | 26999030 | 5.47 | 4 | 74.0007 | 3.94(98.48%) | 3.79(94.73%) | 42.91 | Module |
| XL5C06 | 41118536 | 37554848 | 6.17 | 5.59 | 91.3331 | 5.51(98.68%) | 5.33(95.36%) | 48.09 | Module |
| XL5C07 | 47994290 | 37399786 | 7.2 | 5.56 | 77.9255 | 5.48(98.64%) | 5.30(95.30%) | 48.54 | Module |
| XL5C08 | 41770194 | 32246720 | 6.27 | 4.79 | 77.2003 | 4.71(98.51%) | 4.54(94.94%) | 46.85 | Module |
| XL5C09 | 42121628 | 32292380 | 6.32 | 4.8 | 76.6646 | 4.74(98.67%) | 4.58(95.37%) | 49.04 | Module |
| XL5C10 | 40388550 | 34696144 | 6.06 | 5.16 | 85.9059 | 5.10(98.73%) | 4.93(95.54%) | 48.18 | Module |
| XL5C11 | 40147408 | 34247078 | 6.02 | 5.09 | 85.3033 | 5.01(98.49%) | 4.83(94.90%) | 48.6 | Module |
| XL5C12 | 44727500 | 36405554 | 6.71 | 5.41 | 81.3941 | 5.34(98.69%) | 5.17(95.46%) | 49.05 | Module |
| XL5C13 | 48623102 | 43628984 | 7.29 | 6.48 | 89.7289 | 6.39(98.58%) | 6.17(95.09%) | 48.6 | Module |
| XL5C14 | 52311974 | 44483734 | 7.85 | 6.61 | 85.0355 | 6.52(98.63%) | 6.30(95.22%) | 48.95 | Module |
| XL5C15 | 44520554 | 32911994 | 6.68 | 4.88 | 73.9254 | 4.82(98.61%) | 4.65(95.21%) | 47.57 | Module |
| XL5C16 | 54300100 | 47049624 | 8.15 | 7 | 86.6474 | 6.90(98.64%) | 6.67(95.26%) | 48.63 | Module |
| XL5C17 | 44261488 | 40511044 | 6.64 | 6.03 | 91.5266 | 5.94(98.60%) | 5.73(95.11%) | 47.78 | Module |
| XL5C18 | 62877702 | 49540340 | 9.43 | 7.37 | 78.7884 | 7.27(98.76%) | 7.04(95.61%) | 48.77 | Module |
| XL5C19 | 45754822 | 40182310 | 6.86 | 5.97 | 87.8209 | 5.89(98.62%) | 5.69(95.20%) | 48.28 | Module |
| XL5C20 | 58572212 | 49433004 | 8.79 | 7.34 | 84.3967 | 7.24(98.55%) | 6.98(95.02%) | 48.45 | Module |
| XL5N01 | 46220644 | 32901774 | 6.93 | 4.89 | 71.1842 | 4.82(98.66%) | 4.66(95.34%) | 48.16 | Control |
| XL5N02 | 47654932 | 31068556 | 7.15 | 4.61 | 65.1948 | 4.55(98.71%) | 4.40(95.44%) | 45.94 | Control |
| XL5N03 | 47311908 | 37274772 | 7.1 | 5.54 | 78.7852 | 5.46(98.63%) | 5.27(95.23%) | 47.3 | Control |
| XL5N04 | 51704322 | 42714810 | 7.76 | 6.35 | 82.6136 | 6.26(98.58%) | 6.04(95.12%) | 48.52 | Control |
| XL5N05 | 44589824 | 38305382 | 6.69 | 5.69 | 85.9061 | 5.61(98.58%) | 5.41(95.07%) | 47.49 | Control |
| XL5N06 | 43496658 | 30759590 | 6.52 | 4.57 | 70.7171 | 4.51(98.69%) | 4.35(95.33%) | 44.31 | Control |
| XL5N07 | 49323228 | 39069856 | 7.4 | 5.81 | 79.2119 | 5.73(98.63%) | 5.53(95.23%) | 47.78 | Control |
| XL5N08 | 47279314 | 38285846 | 7.09 | 5.69 | 80.978 | 5.62(98.76%) | 5.44(95.62%) | 48.47 | Control |
| XL5N09 | 37576922 | 17050762 | 5.64 | 2.52 | 45.3756 | 2.49(98.57%) | 2.40(95.14%) | 47.73 | Control |
| XL5N10 | 43212292 | 36166114 | 6.48 | 5.37 | 83.694 | 5.30(98.56%) | 5.11(95.06%) | 48.37 | Control |

**Table S3** The mapping rate of all samples

| **Sample** | **Reads Number** | **Uniquely mapped reads** | **Mapped unique** | **Mapped multi** | **Mapped multi+** | **Unmapped MM** | **Unmapped short** | **Unmapped other** |
| --- | --- | --- | --- | --- | --- | --- | --- | --- |
| XL5C01 | 14074804 | 11063878 | 78.61% | 2761037(19.62%) | 18918(0.13%) | 43600(0.31%) | 180892(1.29%) | 6479(0.05%) |
| XL5C02 | 15585993 | 13673297 | 87.73% | 1732475(11.12%) | 32232(0.21%) | 18013(0.12%) | 122929(0.79%) | 7047(0.05%) |
| XL5C03 | 17089295 | 15015317 | 87.86% | 1910213(11.18%) | 29247(0.17%) | 21752(0.13%) | 98613(0.58%) | 14153(0.08%) |
| XL5C04 | 19299941 | 17418104 | 90.25% | 1709136(8.86%) | 25844(0.13%) | 34286(0.18%) | 92539(0.48%) | 20032(0.1%) |
| XL5C05 | 13499515 | 10699148 | 79.26% | 2601434(19.27%) | 21152(0.16%) | 25585(0.19%) | 139654(1.03%) | 12542(0.09%) |
| XL5C06 | 18777424 | 16700528 | 88.94% | 1899259(10.11%) | 19974(0.11%) | 34107(0.18%) | 115712(0.62%) | 7844(0.04%) |
| XL5C07 | 18699893 | 16986077 | 90.84% | 1473443(7.88%) | 31599(0.17%) | 25019(0.13%) | 171469(0.92%) | 12286(0.07%) |
| XL5C08 | 16123360 | 13868816 | 86.02% | 2102491(13.04%) | 17335(0.11%) | 15599(0.1%) | 115920(0.72%) | 3199(0.02%) |
| XL5C09 | 16146190 | 14192688 | 87.90% | 1746945(10.82%) | 15547(0.1%) | 11043(0.07%) | 176913(1.1%) | 3054(0.02%) |
| XL5C10 | 17348072 | 15661637 | 90.28% | 1564765(9.02%) | 20502(0.12%) | 21790(0.13%) | 71562(0.41%) | 7816(0.05%) |
| XL5C11 | 17123539 | 15273844 | 89.20% | 1623954(9.48%) | 26460(0.15%) | 39406(0.23%) | 149701(0.87%) | 10174(0.06%) |
| XL5C12 | 18202777 | 15382134 | 84.50% | 2655507(14.59%) | 16868(0.09%) | 20849(0.11%) | 119040(0.65%) | 8379(0.05%) |
| XL5C13 | 21814492 | 19445333 | 89.14% | 2122748(9.73%) | 37866(0.17%) | 31216(0.14%) | 159737(0.73%) | 17592(0.08%) |
| XL5C14 | 22241867 | 19738846 | 88.75% | 2292008(10.3%) | 34001(0.15%) | 25293(0.11%) | 126116(0.57%) | 25603(0.12%) |
| XL5C15 | 16455997 | 12608529 | 76.62% | 3450351(20.97%) | 12580(0.08%) | 84091(0.51%) | 296563(1.8%) | 3883(0.02%) |
| XL5C16 | 23524812 | 20997834 | 89.26% | 2335730(9.93%) | 27951(0.12%) | 30638(0.13%) | 121836(0.52%) | 10823(0.05%) |
| XL5C17 | 20255522 | 18156683 | 89.64% | 1948637(9.62%) | 26077(0.13%) | 18171(0.09%) | 92908(0.46%) | 13046(0.06%) |
| XL5C18 | 24770170 | 22234148 | 89.76% | 2302346(9.29%) | 38754(0.16%) | 29656(0.12%) | 147613(0.6%) | 17653(0.07%) |
| XL5C19 | 20091155 | 17883929 | 89.01% | 2009538(10%) | 31225(0.16%) | 15160(0.08%) | 142256(0.71%) | 9047(0.05%) |
| XL5C20 | 24716502 | 22145990 | 89.60% | 2323249(9.4%) | 35522(0.14%) | 18559(0.08%) | 180818(0.73%) | 12364(0.05%) |
| XL5N01 | 16450887 | 14781378 | 89.85% | 1512996(9.2%) | 22642(0.14%) | 24448(0.15%) | 99784(0.61%) | 9639(0.06%) |
| XL5N02 | 15534278 | 12831357 | 82.60% | 2534193(16.31%) | 18981(0.12%) | 19815(0.13%) | 126179(0.81%) | 3753(0.02%) |
| XL5N03 | 18637386 | 16456196 | 88.30% | 1807229(9.7%) | 18067(0.1%) | 24221(0.13%) | 322687(1.73%) | 8986(0.05%) |
| XL5N04 | 21357405 | 19392064 | 90.80% | 1769032(8.28%) | 28634(0.13%) | 35099(0.16%) | 107684(0.5%) | 24892(0.12%) |
| XL5N05 | 19152691 | 17341125 | 90.54% | 1669203(8.72%) | 19186(0.1%) | 23233(0.12%) | 89571(0.47%) | 10373(0.05%) |
| XL5N06 | 15379795 | 11838990 | 76.98% | 3394181(22.07%) | 15813(0.1%) | 16197(0.11%) | 112056(0.73%) | 2558(0.02%) |
| XL5N07 | 19534928 | 17554481 | 89.86% | 1837966(9.41%) | 19210(0.1%) | 21028(0.11%) | 95108(0.49%) | 7135(0.04%) |
| XL5N08 | 19142923 | 17400240 | 90.90% | 1594658(8.33%) | 20828(0.11%) | 25188(0.13%) | 90503(0.47%) | 11506(0.06%) |
| XL5N09 | 8525381 | 7463971 | 87.55% | 927885(10.88%) | 14885(0.17%) | 25882(0.3%) | 88969(1.04%) | 3789(0.04%) |
| XL5N10 | 18083057 | 16506501 | 91.28% | 1416099(7.83%) | 21434(0.12%) | 23961(0.13%) | 106586(0.59%) | 8476(0.05%) |
